# Fetal Goiter Diagnosed in a Euthyroid Patient: An Unusual Presentation of the Fetal Thyroid Disease

**DOI:** 10.7759/cureus.41483

**Published:** 2023-07-06

**Authors:** Carolina Parra Meza, Martha L Africano León, Natalia Quintero Reyes, Silvia N Suarez Mantilla, Claudia Patricia Alvarez Orduz

**Affiliations:** 1 Gynecology and Obstetrics, Hospital Universitario de Santander, Bucaramanga, COL; 2 Maternal and Fetal Medicine, Universidad Industrial de Santander, Bucaramanga, COL; 3 Pediatrics, Clinica San Luis, Bucaramanga, COL; 4 Neonatology, Universidad Industrial de Santander, Bucaramanga, COL; 5 Gynecology and Obstetrics, Universidad Autónoma de Bucaramanga, Bucaramanga, COL; 6 Pediatrics, Hospital Universitario de Santander, Bucaramanga, COL; 7 Pediatrics, Universidad Industrial de Santander, Bucaramanga, COL; 8 Gynecology, Hospital Universitario de Santander, Bucaramanga, COL

**Keywords:** hypothyroidism, intrauterine treatment, euthyroid, fetal, goiter

## Abstract

Fetal thyroid disease is rare, and the disease is mostly contextualized in the setting of a treated maternal thyroid disease. The presentation of thyroid disease in the fetus of a euthyroid mother is unusual.

This paper presents the case of a 21-week pregnant woman with an incidental finding from a detailed anatomy ultrasound and evaluates available diagnostic and therapeutic management options.

There is no consensus with sufficient evidence given the unusual presentation of this type of pathology. In most cases, the evidence is in the etiology of a mother with previous thyroid pathology that modifies the fetal outcome. Hence, it is important to describe cases to accumulate and, at some point, sufficient evidence of different treatments, with the intention of improving the quality of the recommendations.

The management of fetal euthyroid goiter is a complex challenge. Most specialists manage the information on a case-by-case basis, with the same general goals as in patients with other thyroid pathologies.

## Introduction

Fetal thyroids mature at approximately 12 weeks of gestation, but active secretion of hormones does not occur before the middle of the second trimester, at around 17 weeks [1]. A fetal goiter is any increase greater than two standard deviations above the 95th percentile. It is a rare pathology and is present in one in 40,000 pregnancies [2].

Fetal thyroid goiters are initially diagnosed by ultrasound, most effectively in the second or third trimester. Mothers with known thyroid disorders should be carefully examined.

This paper presents the unusual finding of a fetal goiter that was detected by prenatal ultrasound in a euthyroid mother and its postnatal outcome. It also reviews the main causes of fetal goiter, a pathology that is infrequent and easily detectable in an initial study such as a control ultrasound.

## Case presentation

**Figure 1:**
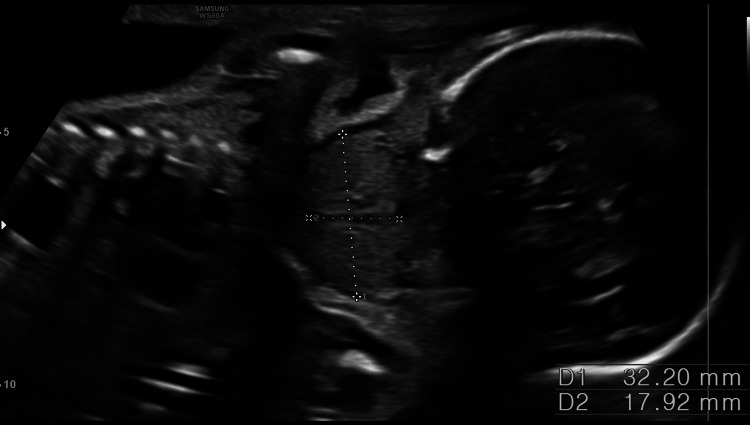
Coronal view of the 21.1-week fetus with a visible thyroid goiter on the anterior face

**Figure 2:**
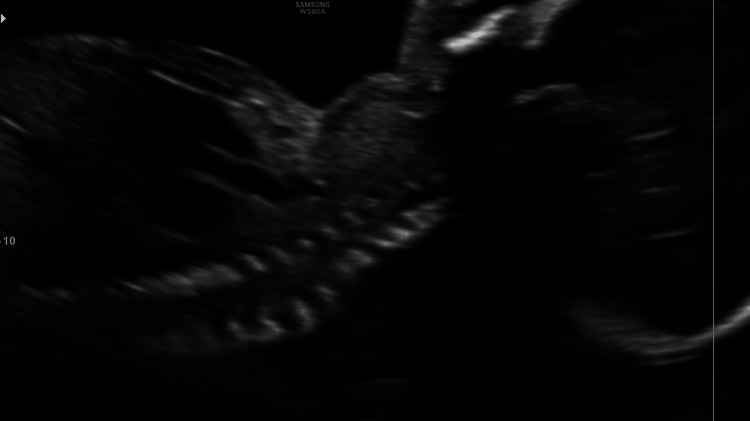
Sagittal view of the 21.1-week fetus with a visible thyroid goiter on the anterior face

**Figure 3:**
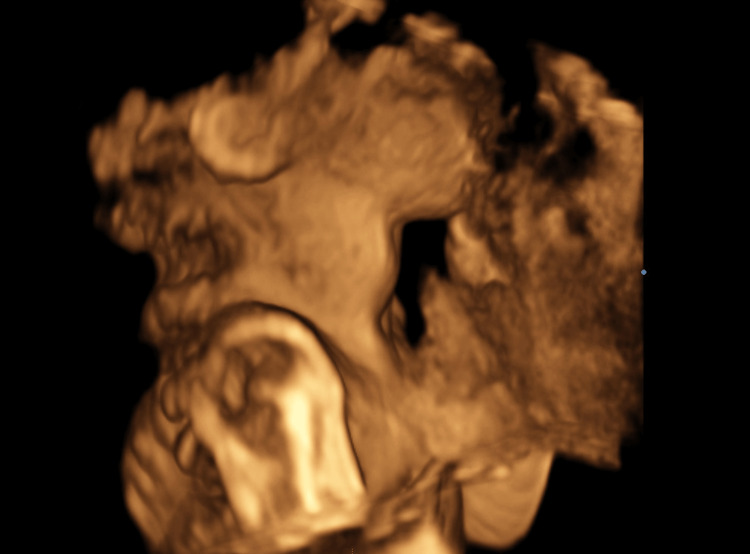
A 3D reconstruction of the 21.1-week fetus with a visible thyroid goiter on the anterior face

**Figure 4:**
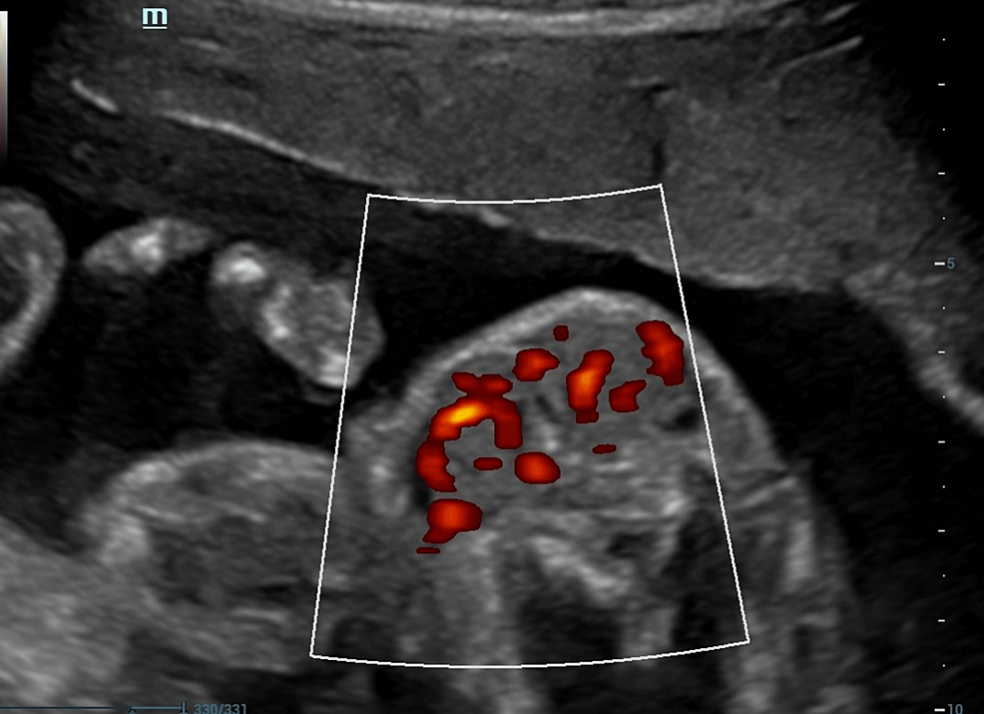
Color Doppler of the fetal goiter

A 35-year-old patient, G3P2V2, with no significant history, was undergoing routine prenatal care. During a detailed prenatal ultrasound, the doctor observed incidental evidence of a homogeneous mass in the anterior part of the neck of the fetus with a diameter of 18 x 15 mm (greater than the 97.5 percentile for age). Figures 1, 2, 3 show the 21-week-old fetus with a mass that is compatible with fetal goiter, without other reported anatomical alterations, and with a Doppler pattern compatible with hypothyroid goiter (Figure 4)

**Figure 5:**
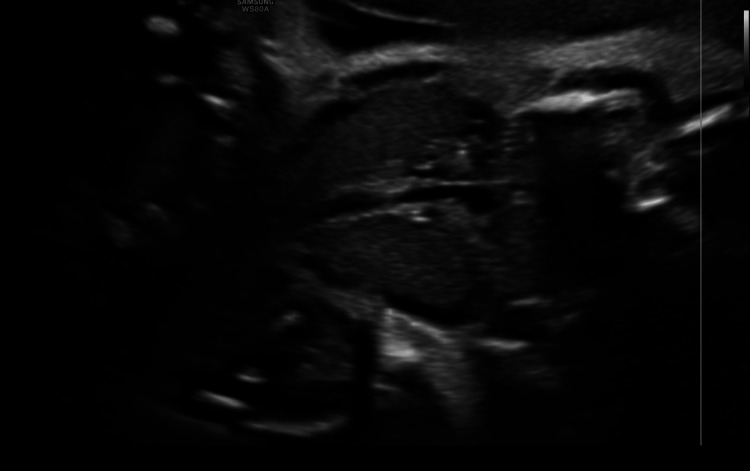
Fetal goiter at week 33

Because of this finding, a maternal thyroid profile was requested. The profile showed a slight decrease in T4 L (0.85/0.90), with a normal thyroid-stimulating hormone (TSH) of 2.6 mIU/L and negative antiperoxidase antibodies. Monitoring was performed every two weeks by ultrasound, without significant changes in the findings. After week 27, at the perinatal meeting, a therapeutic trial with levothyroxine 25 mcg was given orally to the mother, reducing her TSH levels to 2.15 mIU/L. In week 33, a new ultrasound control was performed, showing a 34 x 27 mm fetal goiter, without evidence of polyhydramnios or other signs suggesting extrinsic compression (Figure 5).

During prenatal follow-up, the mother was offered a management choice involving cordocentesis to measure fetal TSH, but she did not accept this because of the potential risks of the procedure and because of the non-availability of levothyroxine in ampoules in Colombia, which would be the treatment if it was required. During subsequent check-ups, the fetus remained stable, and the baby was born via cesarian due to the risk of fetal complications associated with the size of the fetal goiter. However, an ex utero intrapartum treatment (EXIT), a technique used to secure the airway at the time of cesarean section before the baby is separated from the mother, was not necessary.

A term fetus was delivered weighing 3,025 grams, with no change in neonatal adaptation. Follow-up of the newborn showed only a grade II goiter and altered umbilical cord TSH (287.44 µUI/ml). Fetal blood chemistry reported a normal total and free T4 and total and free T3; therefore, thyroglobulin and levothyroxine supplementation was reduced to 50 µg/day. Discharge was allowed on the third day of life with active supplementation.

## Discussion

Pathophysiology

In intrauterine life, the thyroid is the first endocrine gland to be seen on ultrasound. It reaches its final position at approximately seven weeks of gestation, and from week 12, it begins to gain weight rapidly. However, the secretion of functional thyroid hormone does not begin until 16 to 17 weeks of gestation [3]. Its ability to concentrate iodine in vivo appears in weeks 12-14. Therefore, before the first 12 weeks, the fetus depends exclusively on the maternal synthesis of the thyroid hormone to achieve adequate neurological development. Maternal TSH does not cross the placenta [4].

The development of the pituitary gland at weeks 18-22 results in a rapid increase in TSH production, which leads to an increase in levels of circulating T4 and a temporary decrease in T3 which is reversed in the third semester. These hormones have an impact on the functions and structures of the fetal nervous system [5].

There are two types of thyroid receptors whose dysfunction has been found responsible for generating fetal goiter: one is the thyroid-stimulating antibody receptor and the other is the blocking thyroid receptor. The first generates hyperthyroidism and the second causes hypothyroidism [4].

It is recognized that alterations in the fetal thyroid can generate mechanical (due to the mass effect) and biochemical alterations. Mechanical complications include polyhydramnios and asphyxia or dystocia at the time of delivery. Esophageal and tracheal obstructions may also occur during pregnancy, depending on the size of the mass [5].

The biochemical effects depend on the cause of the goiter, whether it is hypothyroidism or hyperthyroidism. In hyperthyroidism, heart failure, decreased growth, and mental retardation can occur. In hypothyroidism, cognitive and motor alterations may occur, as well as an acceleration of bone maturation, craniosynostosis, and stillbirth secondary to heart failure or thyrotoxicosis [1,5].

According to the function of the fetal thyroid gland, it can be classified as hypothyroid, hyperthyroid, or euthyroid goiter. The measurement of TSH in the cord is the gold-standard method for diagnosing and classifying thyroid hormone function at the fetal level [6].

The actual neonatal TSH value is obtained seven days after birth. However, for more than 50 years, doctors have suggested and performed a TSH screening of the umbilical cord at the time of birth to identify thyroid disease in the newborn [5].

In general, one in 4,000 newborns has congenital hypothyroidism, the main cause of which is the use of antithyroid medications or excessive iodine intake during pregnancy. Usually, this is asymptomatic, and a small percentage present with goiter. In patients with elevated TSH results, measuring free and total T4 is recommended, in addition to a second screening between two and four weeks of life [7].

Intrauterine diagnosis

The diagnosis of fetal goiter can be made by means of detailed anatomy ultrasound, which is performed in the second and third trimesters. The sagittal profile of the fetus allows for the identification of the increase in the volume of the gland. However, there are normograms based on gestational age that allow monitoring. The ultrasound findings are typically related to an anterior mass found in the midline and are symmetrical, homogeneous, and hypervascular [8].

Huel et al. [9] published an interesting study that retrospectively described ultrasound findings in 39 fetuses diagnosed with fetal goiter, which could help in differentiating hypothyroid goiter from that of hyperthyroid origin, for example, by analyzing the vascularization pattern with the use of color Doppler. When this pattern is centralized, it reflects overactivity of the gland, as in Graves’ disease. If the pattern is peripheral, it could be related to the trophic vascularization of a hypertrophic but inactive gland. Another finding that this study described is bone maturation, assessing ossification in the distal portion of the femur. A delay in maturation occurs in hypothyroid goiters, while an acceleration in bone maturation is more frequent in hyperthyroid patients. The presence of fetal tachycardia is an indicator of hyperthyroidism, while an increase in fetal movements has been observed in cases of fetal hypothyroidism [9].

Intrauterine treatment

Once fetal goiter is detected, it is important, if possible, to assess fetal thyroid function percutaneously by cordocentesis [10]. If a hypothyroid origin is confirmed, it can be treated directly with an intra-amniotic administration of the thyroid hormone [11,12]. However, in Colombia, levothyroxine in ampoules is not available for such a procedure. For this reason, and as some case reports have described, treatment must be administered immediately after the baby is born, and rigorous therapeutic follow-up must be continued during postnatal life [13].

This treatment is not universally accepted, and there are no guidelines for it available at the time of the review. A group of authors recommends management only in case of symptoms because the risks associated with fetal cordocentesis include chorioamnionitis and cord rupture. Furthermore, it is not clear how much and how often medication should be administered to achieve adequate control of the expected complications of the diagnosed disease nor when the last dose should be applied to avoid reducing the risk of long-term complications. This is why the treatment decision and the type of treatment should be widely discussed, noting its risks and benefits [14].

## Conclusions

Evidence exists for the diagnosis of fetal goiter from thyroid gland volume normograms, which can be evaluated by flow patterns or findings associated with the development of other fetal characteristics to determine whether the goiter is hyper- or hypothyroid. A diagnosis that the goiter is fetal is obtained by cordocentesis, a procedure that has associated risks and complications. At the moment, the ideal time to start treatment in patients with hypothyroid goiter and how often or when to inject T3 or T4 for the last time is unclear. There is even less evidence for hyperthyroid goiter.

Goiters are normally related to the mother's consumption of drugs that work on the gland, but this is not the case in euthyroid patients, who are less commonly seen. We provide this case description with the intention of describing the findings and management in a country that does not have access to intrauterine management due to the unavailability of intravenous medication and where a clinical follow-up to avoid mechanical complications is the main prenatal follow-up event. In this case, early postnatal management in a high-risk patient meant that the newborn is a healthy infant at the time of publication.
